# Photons as a 21st century reagent

**DOI:** 10.1038/s41467-019-13988-4

**Published:** 2020-02-06

**Authors:** Holly E. Bonfield, Thomas Knauber, François Lévesque, Eric G. Moschetta, Flavien Susanne, Lee J. Edwards

**Affiliations:** 1 Chemical Development, GSK, Gunnels Wood Road, Stevenage, Hertfordshire, SG1 2NY UK; 2 Department of Pure and Applied Chemistry WestCHEM, University of Strathclyde 295 Cathedral Street, Glasgow, Scotland, G1 1XL UK; 3 Medicinal Sciences, Worldwide Research and Development, Pfizer, Inc., Eastern Point Road, Groton, Connecticut 06340 USA; 4 Process Research & Development, Merck & Co., Inc., Rahway, New Jersey 07065 USA; 5 Process Research & Development, AbbVie, Inc., 1401 Sheridan Road, North Chicago, Illinois 60064 USA

**Keywords:** Chemical synthesis, Photocatalysis

## Abstract

A pharmaceutical industry viewpoint on how the fundamental laws of photochemistry are used to identify the parameters required to implement photochemistry from lab to scale. Parameters such as photon stoichiometry and light intensity are highlighted within to inform future publications.

Photochemistry employs photons to drive chemical transformations. Traditional approaches rely on the direct excitation of bonds with light^1,2^. In contrast, photoredox catalysis uses a photosensitizer to facilitate electron transfer, generating reactive intermediates under mild conditions^3^. From a pharmaceutical industry point-of-view, photochemistry is a powerful tool to access high-energy intermediates that can provide novel reactivity and enables new disconnections, allowing target- and diversity-oriented synthesis to be explored^4^. Photochemistry is extensively used within medicinal chemistry projects, but has not yet been extensively employed for the commercial manufacture of pharmaceutical agents^5^.

The scientific community has published new and exciting photochemical methods, but in our experience these transformations can be challenging to reproduce^3,6^. We believe that this difficulty results from an under-emphasized importance of fundamental photochemical concepts, which renders translation of methods between set-ups challenging. Most authors try to describe the key features of the photochemical system used in their experiments; however, the description may not be completely sufficient to ensure reproducibility in a different lab or comparable reactor system. It can be quite common to find little technical detail about the equipment setup in the Supplementary Information and many summaries fall short of fully characterizing the photo-physical properties of their setup. We believe that careful characterization and description of the photochemistry equipment is essential; more systematic and better documented experimentation will enable greater mechanistic understanding, leading to facile identification of the key scale-up factors.

From a review of the fundamentals of photochemistry, we will detail the minimum information to include in any photochemistry-related publication. This will enable the chemistry community to more readily assess and adopt photochemical transformations. We will then look toward the future of photochemistry for a manufacturing system that applies this technology.

## Grotthuss–Draper law

Light must be absorbed by a chemical substance for a photochemical reaction to occur. Photon absorption excites a molecule from its ground state to an excited state. Chemical substances only absorb at specific wavelengths, so it is critical that the right wavelength is selected for the desired transformation.

**Fig. 1:**
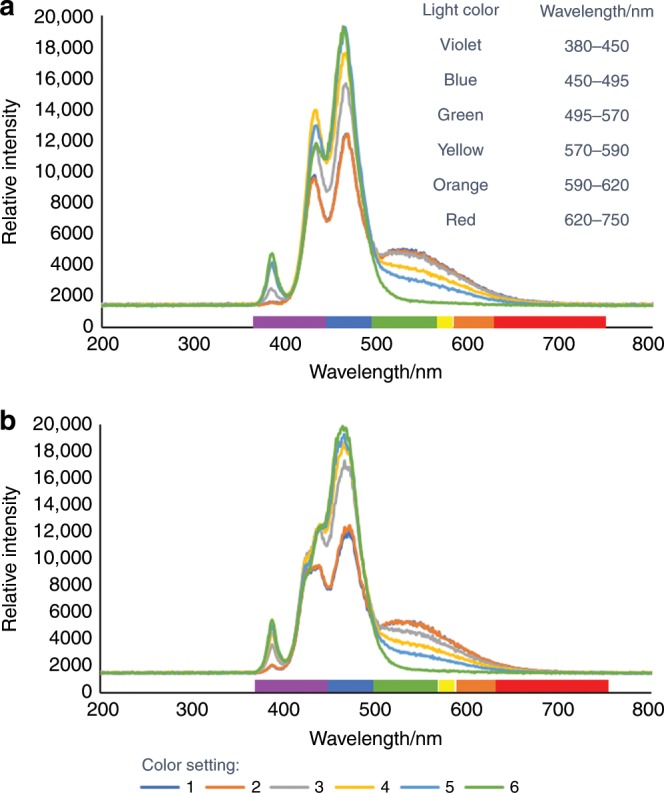
Comparison of a commonly used light source. **a** Spectral output overlays of a 34 W blue LED (A) at intensity setting 10 and color settings 1–6. **b** Spectral output overlays of a 34 W blue LED (B) at intensity setting 10 and color settings 1–6. LED A and B are the same maker and model from different batches; both are emitting from violet to red light. Figure adapted from (10.1002/cptc.201900203). Copyright Wiley-VCH Verlag GmbH & Co. KGaA. Reproduced with permission.

Stating that “blue light-emitting diode (LED) strips”, “a 34 W blue LED”, or “a compact fluorescent light (CFL)” is used for the chemistry does not provide sufficient information about the light’s spectral output. Blue light refers to 450–495 nm. Some blue LED light sources offer the ability to change the color and intensity. These parameters are rarely reported in the literature but can drastically affect the spectral output of the light source and should be included in all publications^7^. Furthermore, these non-descript domestic light sources often suffer from different spectral outputs as a result of batch-to-batch variability (Fig. 1). Thus, any photochemical publication should characterize the spectral output of the light source used. At minimum, the most dominant wavelength in the output should be reported.

**Fig. 2:**
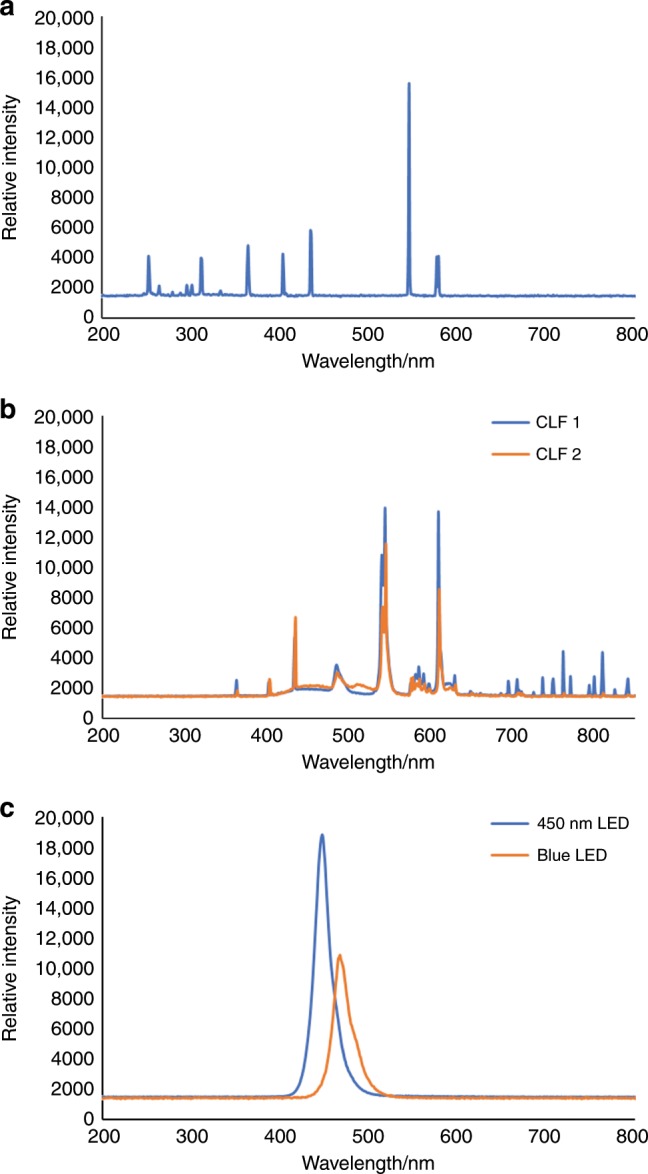
Comparison of the spectral outputs and relative light intensities of commonly used light sources. **a** 12 W medium pressure mercury lamp; **b** two CFLs (overlaid); **c** a 450 nm LED flow reactor, and a blue LED screening platform at 30 mA (overlaid). Figure adapted from (10.1002/cptc.201900203). Copyright Wiley-VCH Verlag GmbH & Co. KGaA. Reproduced with permission.

Other light sources, such as CFLs and medium pressure mercury lamps, are composed of multiple emission bands and most reported blue LEDs emit a broad spectrum (Figs. 1 and 2); hence, it is difficult to define the exact wavelength of interest for the transformation. Polychromatic light sources might be capable of promoting the desired transformation, but reaction mixtures can also absorb other wavelengths that can facilitate unwanted side reactions^8^. Furthermore, emission of undesired wavelengths will lead to unnecessary energy consumption and increase cooling requirements on scale. For any light source used, it must be remembered that temperature and spectral filters affect the spectral output. Differences in spectral output can result in differing yields and/or unexpected side reactions. The current inability to compare light source outputs causes the reproducibility challenges with which we are currently faced.

## Stark–Einstein law or photoequivalence law

One photon activates a single molecule. This law highlights the importance of knowing the photon output of the light source used (i.e., photon equivalents) and the significance of reporting quantum yields.

Actinometers can be used to characterize a light source by using a reaction with a known quantum yield to calculate the number of photons absorbed. A number of actinometry reactions have been reported, but we have failed to identify a simple way to operate the system that covers a wide range of wavelengths^9,10^. With this in mind, we are calling for a procedurally simple homogeneous actinometry reaction that is applicable to both batch and flow photoreactors.

The excited state generated by absorption of a photon can either react to give a photochemical product or decay to the ground state by release of light (luminescence) or heat (vibrational relaxation). Vibrational relaxation can result in increased internal reaction temperatures compared with the surrounding air temperature.

For literature reactions that we have reproduced, we have discovered that, although the air surrounding a reactor can be at ambient temperature, it is not uncommon for the internal reaction temperature to reach 60 °C–80 °C^11^. The specific type of reactor used for a reaction affects the internal reaction temperature^11^. Signifying the reactor configuration and any heat transfer elements used is the key. We advise groups to record the internal reaction temperature for all photochemical reactions.

## Bunsen–Roscoe law

The photochemical effect is directly proportional to the total energy dose, irrespective of the time to deliver the dose (intensity of light × time of exposure = constant).

This often-forgotten law underlines the effect that light intensity has on reaction kinetics. Changing between systems/lamps can lead to differences in reaction rate. For example, on scaling from a blue LED screening platform with a relative light intensity of ~11,000 to a 450 nm LED flow reactor, the light intensity increases to ~18,000 and so the reaction rate often increases (Fig. 2).

## Inverse-square law of light

Light intensity decreases with distance from the light source. Many publications state that the reaction mixture is placed “approximately” *X* cm away from the light source. However, this law crudely enables modulation of the light intensity by varying the distance of the reaction mixture from the light source, which is a fundamental principle of photochemistry (Bunsen–Roscoe law). Therefore, to be able to accurately reproduce a photochemical reaction, it is vital that the exact distance between the light source and the reaction mixture is provided.

## Beer–Lambert law

This law relates the attenuation of light to the properties of the material through which the light is travelling. This is also something important to take into consideration and will be linked to the path length, the concentration of the photocatalyst, and its molar absorption coefficient. It would be very useful to have better descriptions of these parameters: in particular, the molar absorption coefficients of catalysts at the wavelength of the light source being used or at least report the UV-Visible spectrum. One approach that has been used to great effect is to use the molar absorption coefficient to design the path length and catalyst loading in a photochemical reaction.

One way to think of a photon is as a reagent. Therefore, just like any reagent, we should know how many photon equivalents are added to our reaction mixtures. Information on the light source, photon stoichiometry, internal reaction temperature, light intensity, distance between the light source and reaction mixture, and path length are all key scale-up factors^8^.

Many pharmaceutical companies and external technology suppliers have developed their own photochemistry platforms, meaning that there is a trend towards standardization within companies^11–13^. Characterization of equipment within each organization/group is the first step toward the future of photochemistry in the modern era. Current state-of-the-art industrial systems allow for identical LEDs (i.e., scientific grade LEDs ± 20 nm) of specific pseudo-monochromatic wavelengths or LASERs to be used, light intensity to be modulated, and reaction scale-up from screening to batch to flow, to be performed in a facile, modular manner, enabling more detailed photoreactor characterization.

In the future, we envisage fully characterized photochemical platforms within organizations where all scale-up factors are digitally monitored to drive the generation of consistent data sets.

## Additional considerations for scaling

For production scale, in addition to the aforementioned scale-up factors discussed, we also need to understand additional parameters.

### Mixing requirements

Mixing studies are required for both batch and flow reactors, as residence times and reaction times depend on mass transfer. Reynolds and Bodenstein numbers for flow photoreactors should be calculated and the authors should state explicitly whether the flow is laminar or turbulent^14^.

### Intensity–space definition

Knowing the actual illuminated surface area on a smaller system can then be translated to a large-scale reactor by upscaling the surface area to photon ratio. (intensity based on space). We have found that the angle of the LED lens affects this parameter^11^.

### Solids

Some reactions require heterogeneous reagents. These fall foul of the Beer–Lambert law at any scale. Heterogeneous mixtures are challenging to handle in traditional plug flow. This can be somewhat mitigated by using cascaded continuous stirred tank reactors or a Dart reactor. In general, multiphase photochemical reaction engineering is one of many (likely) logical paths forward in the field.

## Final remarks and future outlook

In our experience, depending on the kinetics, some photochemical reactions are suited to flow, whereas other reactions, with longer residence times, require a batch setup, negating the possibility of having one universal photoreactor^15^. Instead, we believe that the future of photoreactor design will be in collaboration between academics looking at the photo-physics of a transformation and engineers using the kinetic data generated to inform reactor design, generating bespoke reactors for a specific process.

For any publication, we expect a description of the reactor characteristics, identified as key parameters through the laws of photochemistry. With these parameters, it is our belief that the transfer of reactions between set-ups will be simplified for all chemists. Hence, we implore all scientists and manufacturers of commercial equipment to fully characterize their systems. In so doing, we strongly believe that a better characterization of photochemical reactors and better understanding of photochemical processes will lead to a broader usage of photochemistry across academia and industry.
